# Explainable CAD System for Classification of Acute Lymphoblastic Leukemia Based on a Robust White Blood Cell Segmentation

**DOI:** 10.3390/cancers15133376

**Published:** 2023-06-27

**Authors:** Jose Luis Diaz Resendiz, Volodymyr Ponomaryov, Rogelio Reyes Reyes, Sergiy Sadovnychiy

**Affiliations:** 1 Instituto Politecnico Nacional, Escuela Superior de Ingenieria Mecanica y Electrica–Culhuacan, Av. Sta. Ana 1000, Mexico City 04440, Mexico; jdiazr2100@alumno.ipn.mx (J.L.D.R.); rreyesre@ipn.mx (R.R.R.); 2 Instituto Mexicano del Petroleo, Eje Central Lazaro Cardenas Norte 152, Mexico City 07730, Mexico; ssadovny@imp.mx

**Keywords:** acute lymphoblastic leukemia, deep-learning, XAI, nuclei segmentation, leukemia classification

## Abstract

**Simple Summary:**

Leukemia is a type of cancer that affects white blood cells and can lead to serious health problems and death. Diagnosing leukemia is currently performed through a combination of morphological and molecular criteria, which can be time-consuming and, in some cases, unreliable. Computer-aided diagnosis (CAD) systems based on deep-learning methods have shown promise in improving diagnosis efficiency and accuracy. However, these systems suffer from the “black box problem,” which can lead to incorrect classifications. This research proposes a novel deep-learning approach with visual explainability for ALL diagnoses based on robust white blood cell nuclei segmentation to provide a highly reliable and interpretable classification. The aim is to develop a CAD system that can assist physicians in diagnosing leukemia more efficiently, potentially improving patient outcomes. The findings of this research may impact the research community by providing a more reliable and explainable deep-learning-based approach to blood disorder diagnosis.

**Abstract:**

Leukemia is a significant health challenge, with high incidence and mortality rates. Computer-aided diagnosis (CAD) has emerged as a promising approach. However, deep-learning methods suffer from the “black box problem”, leading to unreliable diagnoses. This research proposes an Explainable AI (XAI) Leukemia classification method that addresses this issue by incorporating a robust White Blood Cell (WBC) nuclei segmentation as a hard attention mechanism. The segmentation of WBC is achieved by combining image processing and U-Net techniques, resulting in improved overall performance. The segmented images are fed into modified ResNet-50 models, where the MLP classifier, activation functions, and training scheme have been tested for leukemia subtype classification. Additionally, we add visual explainability and feature space analysis techniques to offer an interpretable classification. Our segmentation algorithm achieves an Intersection over Union (IoU) of 0.91, in six databases. Furthermore, the deep-learning classifier achieves an accuracy of 99.9% on testing. The Grad CAM methods and clustering space analysis confirm improved network focus when classifying segmented images compared to non-segmented images. Overall, the proposed visual explainable CAD system has the potential to assist physicians in diagnosing leukemia and improving patient outcomes.

## 1. Introduction

Blood disorders are among the most challenging problems in medical diagnosis and image processing, where blood samples can be used to analyze a person’s state of health and diagnose various diseases such as allergies, infections, or cancer. Specifically, one of the most lethal cancers with the highest incidence rate is Leukemia, where malformation of the white blood cells causes serious health problems that can lead to death. Although WBCs are involved in protecting the human body, they are also susceptible to illness. The most critical pathological conditions of the white blood cells are blood cancers. As a consequence of malignant mutations in the lymphoid or myeloid cells, there is an uncontrolled proliferation of malformed cells that do not function correctly in the organism, causing a decrease in the patient’s health and even death. This process of malformation and uncontrolled reproduction of white blood cells is called Leukemia [1,2].

Leukemia can be classified according to the type of malignant cell, either lymphoid or myeloid, or the speed of symptoms development, chronic or acute. Acute Lymphoblastic Leukemia (ALL) is the most common during childhood, and due to genetic factors, the most affected ethnicity worldwide by ALL is the Hispanic population [2]. Currently, the way to diagnose Leukemia is based on a mixture of morphological and molecular criteria. The morphological classification relies on the FAB (French-American-British) medical criteria, established on recognizing characteristics or patterns such as the number of white blood cells, shape, and size, among others, where it is possible to differentiate between the types [2,3].

One major disadvantage of this procedure is the time consumption for the specialist in the analysis of each sample and the reliability of the diagnosis [4]. In addition, in low-income countries where health systems are overwhelmed, the time to find an appointment for the performance of these tests is high, which can result in a late diagnosis. Computer Aided Diagnostic (CAD) systems assist physicians in routine tasks to diagnose more efficiently, accurately, and with shorter diagnostic times, providing a better outcome for the patient.

In particular, CAD systems based on Deep Learning methods have recently gained relevance due to the good metrics obtained in research articles. However, as Loddo and Putzu [5] stipulate, many of the systems based on Deep Learning, specifically segmentation and classification systems of blood smear images, need a deeper analysis of the results beyond the metrics and learning curves. One major challenge associated with Deep Learning models is the “Black box problem,” where the lack of semantic associations between input data and predicted classes hinders interpretability. This means that although a Deep Learning model may achieve excellent metrics and accurately classify results, the underlying associations made by the model might be incorrect. This conveys a significant risk when applying these systems to different databases or integrating them into routine clinical practice.

The growing spectrum of diseases and the potential of Computer Diagnosis have sparked intense research into white blood cell (WBC) segmentation and leukemia classification. Propelled by progress in computer vision and Deep Learning, considerable strides have been taken in addressing the challenges intrinsic to WBC nuclei segmentation and leukemia classification [6,7].

Recent research has shown the positive impact of appropriate pre-segmentation on deep-learning classification in medical imaging. The research of Mahbod et al. [8] highlighted improved performance with the correct use of segmentation masks on dermoscopic images, however, when segmentation was applied inaccurately, it resulted in a decrease in model performance. Similarly, Al-masni et al. [9] found that feeding segmented skin lesions into an integrated computer-aided diagnosis (CAD) system resulted in more effective diagnostic classification.

In the context of WBC segmentation, one of the most relevant studies was carried out by Vogado et al. [10], where color space transformations from RGB to CMYK and Lab* were applied, followed by contrast adjustment and median filtering to enhance the image. Leukocytes were highlighted by subtracting the B channel from the M channel. K-means clustering and morphological operations were subsequently employed. Alternatively, Makem and Tiedeu [11] introduced a WBC nucleus segmentation method by leveraging color space transformations, arithmetical operations, and adaptive PCA fusion. Their approach demonstrated excellent performance with Dice Coefficients of 94.75%, 97.06%, and 90.79% on the BloodSeg, CellaVision, and JTSC databases, respectively, validating its effectiveness across diverse datasets. Meanwhile, Mousavi et al. [12] addressed the WBC nucleus segmentation problem by employing a color balancing method based on the color channels means, converting the image to CMYK and extracting the Magenta channel and then segmenting the image. This approach was trained and tested with 985 and 250 images from the Raabin WBC, respectively, obtaining a Dice Coefficient of 95.42%. After, Tavakoli et al. [13] developed a three-step method for WBC nucleus segmentation. Applying color balancing, RGB to HSL and CMYK conversions, and arithmetic operations to enhance nuclei visibility, followed by Otsu filtering for binarization. The method achieved a Dice Coefficient of 96.75% on a subset of 250 images from the Raabin WBC dataset.

Makem et al. [14] proposed a robust WBC segmentation method based on arithmetic operations and the Fourier transform. They segment the WBC using RGB space operations and Otsu thresholding, followed by Fourier-based image enhancement. The K-means algorithm is then applied for nuclei grouping and segmentation. The method achieved high segmentation accuracy on five databases, with Dice Coefficient results ranging from 86.02% to 97.35%. In comparison, Mayala and Haugsøen. [15] proposed a WBC segmentation method based on finding the minima between two local peaks in the image histogram analysis.

Ochoa-Montiel et al. [16] proposed an intermediate approach between handcrafted and deep-learning methods for WBC segmentation and ALL classification. They employ RGB to HSI transformation, Otsu’s segmentation method, and handcrafted feature extraction techniques. Classification is performed using handcrafted approaches and deep-learning methods based on Alexnet and LeNet architectures.

In contrast, a few WBC segmentation schemes are based entirely on the Deep Learning approach. For example, Haider et al. [17] proposed a Deep Learning approach for WBC segmentation, specifically nucleus and cytoplasm segmentation. They introduced two networks, LDS-NET and LDAS-NET, which are modifications of U-NET with additional features such as residual connections. The combination of these features helps retain information and improve accuracy. The approach of Garcia-Lamont et al. [18] proposes six methods for WBC nucleus segmentation: CPNNHSV, CPNRGB (neural network-based), SOMHSV, SOMRGB (Self Organized Maps-based), and VarHSV, VarRGB (based on chromatic variance). This approach has been tested using three different databases with 660 images.

Zhou et al. [19] applied a modified version of U-Net, a well-known Deep Learning method used for segmentation. U-Net++ architecture modifies the plain skip connections for nested and dense skip connections to combine the high-resolution map feature. This algorithm was trained and tested with 989 and 250 images of the Raabin WBC database, respectively, reaching a Dice Coefficient of 97.19%. Similarly, Oktay et al. [20] proposed a new U-Net-based model with attention. This attention gate allows for highlighting relevant features and removing irrelevant ones resulting in better segmentation. The algorithm was implemented with the Raabin WBC dataset, trained and tested with 989 and 250 images, respectively, resulting in a Dice Coefficient of 96.33%. Finally, He et al. [21] enhanced the Faster R-CNN approach with the Mask R-CNN architecture for WBC segmentation. The method improves the segmentation results by introducing a connection between the convolutional feature maps and generating a masked ROI as an attention module.

The review of the state-of-the-art shows that WBC segmentation and Leukemia classification remains an active and evolving research area. In recent years, the significance of model interpretability and explainability has garnered increasing attention in medical diagnosis. Current methodologies encounter challenges regarding robustness and the elucidation of the underlying rationale behind model predictions. Traditional Handcraft approaches often involve intricate and non-intuitive segmentation steps and typically perform worse than AI models. Deep Learning methods, while achieving impressive performance, frequently suffer from the “Black box problem,” difficulting in the reliability of the diagnosis. Thus, there is a pressing need to explore novel techniques prioritizing model interpretability and explainability. In addition, in assisted medical diagnosis systems, the doctor must understand the reasons that lead to a particular Deep Learning classification so that the physician can implement an accurate and reliable hybrid diagnosis.

In this article, we introduce a novel method for leukemia classification using Explainable Artificial Intelligence (XAI) and segmentation techniques. The unique feature of our approach lies in its use of segmentation as a form of ’hard attention’ mechanism, which enhances the classifier’s accuracy and interpretability by targeting the nucleus of white blood cells (WBCs). We demonstrate the robustness of our segmentation method by testing it across multiple databases. To make the network associations more tangible, we use gradient attention maps that visualize the relevance of various regions, considering both the intensity and location of the ’attention’ within the Region of Interest (ROI). By focusing on the WBC nuclei before classification, our proposed method significantly improves the quantitative and qualitative criteria, outperforming classifiers that do not use segmentation. We also compare Deep Learning approaches and demonstrate the superior efficacy of the Mish activation function over the commonly used Rectified Linear Unit (ReLU). Through these findings, we hope to advance the field of leukemia classification by offering an approach that is not only more accurate but also more explainable.

Furthermore, this research has four main sections; Section 2 shows the datasets and the metrics used for evaluation; Section 3 presents the proposed methodology; Section 4 exhibits results as well as the discussion; finally Section 5 summarized scientific contributions of this research.

## 2. Materials

### 2.1. Datasets

This research used six databases with digitalized images of blood or bone marrow samples. A total of 2823 different images were used to test the developed method. The databases employed have different characteristics concerning each other, such as the number and size of white blood cells, image color, saturation, illumination, etc.

- Leukemia Dataset [16] is formed by 651 classified images of Acute Lymphoblastic Leukemia according to FAB classification (217-ALL1, 217-ALL2, 217-ALL3), with dimensions of 256×320 pixels. This dataset is the only one in the state-of-the-art that labels the different types of Acute Lymphoblastic Leukemia with reliability through cytogenetic tests.
- CellaVision [22] is made up of 100 blood samples, and each image has dimensions of 300×300 pixels and a bit depth of 24 bits. This dataset usually consists of a single cell, and the core color is violet, while the background has pinkish and yellowish tints.
- JTSC [22] is made by the Jiangxi Telecom Science Corporation in China. This dataset consists of 300 images of 120×120 pixels containing the GT of the nucleus and cytoplasm for comparative analysis. It contains a wide variability among its samples since there are cells in which the nucleus has a highly saturated coloration, while in others, the nucleus is almost translucent. Furthermore, the image’s background varies from an intense yellow to a pinkish white.
- SMC_ID (Blood_Seg) [23] is composed of 367 images of WBC with a size of 640×480 pixels. Each sample characterizes by the GT of the nucleus, which facilitates its analysis. Commonly, the images that integrate this dataset have a cell nucleus with low color saturation. Additionally, the WBC is located in diverse positions over the image.
- Raabin_WBC [24]. It provides 1145 images of blood samples, with dimensions of 545×545 pixels, where white blood cells are subdivided into 242 lymphocytes, 242 monocytes, 242 neutrophils, 201 eosinophils, and 218 basophils. Each of these 1145 samples also contains a ground truth, both whole cell and nucleus. This is one of the best databases by now, as it has numerous samples of different cell types classified and annotated with ground truth for analysis and comparison of results.
- ALL_IDB2 [25]. It consists of 260 images of 257×257 pixels. This dataset derives from the ALL-IDB1 dataset, where individual cells have been cropped to obtain the region of interest.

**Figure 1:**
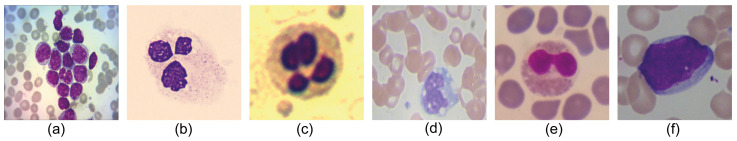
Used dataset images: Leukemia Dataset (**a**), CellaVision (**b**); JTSC (**c**); BloodSeg (**d**); Raabin_WBC (**e**); ALL_IDB2 (**f**).

Examples of the datasets used in the research are shown in Figure 1.

### 2.2. Metrics

For evaluating the proposed segmentation and classification method, seven of the most widely used metrics were employed [26]:

- **Accuracy** value measures the appropriate classification over the total elements.
  (1) $$Acc=\frac{T_{P}+T_{N}}{T_{P}+T_{N}+F_{P}+F_{N}}\;.$$
- **Precision** metric estimates the number of elements correctly classified among all the positive elements to evaluate.
  (2) $$Pre=\frac{T_{P}}{T_{P}+F_{P}}\;.$$
- **Recall** also known as sensitivity, is used to denote the number of positive elements that are correctly classified.
  (3) $$Rec=\frac{T_{P}}{T_{P}+F_{N}}\;.$$
- **Specificity** measures the proportion of true negatives that are successfully identified by the model.
  (4) $$Spec=\frac{T_{N}}{T_{N}+F_{P}}\;.$$
- **Dice Similarity Coefficient** or DSC can be considered to be a harmonic mean of precision and recall. Furthermore, known as F1-Score.
  (5) $$DSC=2*\frac{Precision*Recall}{Precision+Recall}\;.$$
- **Intersection over Union** also known as Jaccard Index is the most important metric in image segmentation tasks since it measures the magnitude of overlap between the GT and the segmented image.
  (6) $$IoU=\frac{T_{P}}{T_{P}+F_{P}+F_{N}}\;.$$

were **TP** represents true positives, **TN** true negatives, **FP** false positives and **FN** false negatives.

## 3. Proposed Method

**Figure 2:**
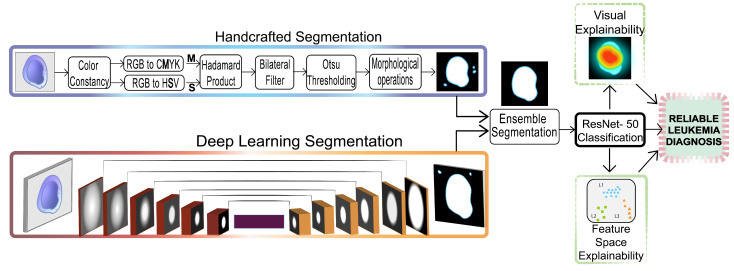
Proposed Method.

In this work, a novel CAD system for Acute Lymphoblastic Leukemia classification was developed. The novel approach relies on an ensemble state of art white blood cell segmentation that acts as a hard attention mechanism for the network, increasing diagnostic accuracy and reliability. Furthermore, a visual Grad CAM interpretation with four gradient activations maps (GradCAM, GRADCAM++, Hi-Res-CAM, Xgrad-CAM) and a clustering space analysis increase the reliability of the method. The proposed system diagram is shown in Figure 2. Below, each of the three phases of the method is presented.

### 3.1. Handcrafted WBC Nuclei Segmentation

This research proposes a new robust and consistent segmentation method for differentiating the WBC nucleus from the rest of the sample. To address the issue of color variations between blood samples, caused by factors such as illumination, microscope type, and staining, a method proposed by Hedge et al. [27] is employed. This method involves multiplying the original RGB channels by a weight calculated based on the ratio between the average grayscale intensity and the average intensity of the respective channel (Red, Green, and Blue), as can be seen below in Equation (7). By applying this approach, the colors in the samples are homogenized, enhancing the tonal consistency across different datasets and improving the method’s applicability and robustness.

(7) $$CC_{Channel}=Channel\;Intensity\left(\frac{mean\;Grayscale}{mean\;Channel\;Intensity}\right)\;.$$

We enhance the WBC nucleus by matching image tonalities and employing color space transformations (RGB to CMYK and HSV). Guided by purple tonalities and high saturation in the ROI region, the Saturation and Magenta channels are combined using the Hadamard product to highlight nuclei and remove unwanted elements. The bilateral filter [28] is employed after the Hadamard product to refine the segmentation process further to eliminate image noise and blur the WBC nucleus. This step ensures that any regions potentially lost during the Hadamard product operation are recovered while maintaining the original shape and integrity of the cell edges. The grayscale image is then transformed to a binary image via the adaptive Otsu Thresholding [29], resulting in an image where the WBC nuclei are highlighted in white and the other components of the image in black.

Since areas with holes could be found in the nucleus of the binarized image, the morphological transformations of closing and filling holes are applied to improve the segmentation process. The closing eliminates the small black regions, filling holes operation dilates the white regions within the WBC nucleus. Finally, a filter by ROI pixel area removes small spurious elements that remain, where the elements with a smaller area of pixels than those established by the threshold are eliminated from the image. All the presented steps of the WBC nuclei segmentation method can be summarized in Algorithm 1.
**Algorithm 1** Proposed Handcrafted WBC Nuclei Segmentation.1:Read RGB image2: CC_RGB←ApplyColorConstancytoRGBimage3: $CMYK_{Image}\leftarrow Transform\;CC\_RGB\;to\;CMYK$4: $M\leftarrow split(CMYK_{Image})$5: $HSV_{Image}\leftarrow Transform\;CC\_RGB\;to\;HSV$6: $S\leftarrow split(HSV_{Image})$7: $Mult_{Image}\leftarrow M⊙S\;\left(Hadamard\;Product\right)$8: $Bilateral_{Image}\leftarrow BilateralFilter\left(Mult_{Image}\right)$9: $Binarized_{Image}\leftarrow Th\_Otsu\left(Bilateral_{Image}\right)$10: $Binarized_{Image}\leftarrow Closing\left(Binarized_{Image}\right)$11: $Binarized_{Image}\leftarrow Fill\_Holes\left(Binarized_{Image}\right)$12: $AreaFilter_{Image}\leftarrow Binarized_{Image}>=pixel\;number$13: $Segmented_{Image}\leftarrow Mask\left(AreaFilter_{Image},RGB\;Image\right)$

### 3.2. Deep Learning WBC Nuclei Segmentation

The encoder-decoder architecture, U-Net [30], was implemented for the Deep Learning segmentation phase. The encoder downsamples the input image and extracts high-level features, while the decoder upsamples the features to reconstruct the original image size and generate a segmentation map. The skip connections between the encoder and decoder help to preserve spatial information and enable precise segmentation of objects. Our implementation of the UNet model has the following structure: Designed for 2-dimensional spatial inputs, begins with an input of 3 channels. The model progresses through five distinct levels, each corresponding to a different size of the channel, expanding from 32 to 512. At each level, the model performs downsampling using strided convolutions, with strides of 1 at the first level, and 2 at subsequent levels. The model employs Instance Normalization, includes a dropout rate of 0.5 for regularization, and uses the Mish activation function for non-linearity [31] (see Equation (8))

(8) $$Mish\left(x\right)=x*tanh(ln\left(\left(1+e^{x}\right)\right)\;.$$

Furthermore, we used the state-of-the-art Unified Focal Loss function [32] as a loss function for our UNet-based model, which can improve the segmentation due to its better handling of class imbalance and the combination of Focal Loss, Equation (9), (distribution-based loss) and Tversky Loss, Equation (10), (region-based loss).

(9) $$L_{mFocalLoss}=\delta {\left(1-p\right)}^{1-\gamma }*L_{BinaryCrossEntropy}\;.$$

(10) $$L_{mFocalTverskyLoss}=\sum_{c=1}^{C}{\left(1-mTI\right)}^{\gamma }\;.$$

(11) $$L_{Unfied\;Focal\;Loss}=\lambda L_{mFocalLoss}+\left(1-\lambda \right)L_{mFocalTverskyLoss}\;.$$

For Unified Focal Loss, see Equation (11), the three tuning parameters are defined as: δ controls the relative weighting of positive and negative classes, γ manages the suppression of background classes and the attention of rare classes, and lastly, λ handles the weights between the distribution-based loss and the region-based loss.

### 3.3. Ensemble Segmentation

Ensemble segmentation is a technique for improving the accuracy and robustness of image segmentation using multiple segmentation models. Combining the predictions of several models can improve the overall performance of the segmentation. The novel method employs a Hybrid ensemble segmentation technique. By combining the proposed Handcrafted and Deep Learning segmentations, we can overcome the limitations of individual approaches and produce more reliable segmentation results. Since the biggest problem in both segmentation methods was the false positives, the logical AND operation was used to merge both masks, significantly reducing the number of false positives and increasing the stability of the ensemble segmentation. For instance, when one of the two methods does not correctly remove a non-ROI region and the other does, this non-ROI region is removed from the Ensemble segmentation mask. After the fusion technique, we applied an area opening as a post-processing operation.

(12) EnsembleMask=HandcraftedMask∧DeepLearningMask.

### 3.4. ALL Classification

In this study, the proposed classifier is based on ResNet-50 [33], which through their residual connections, allows a better back-propagated gradient flow through the network, contributing simultaneously to assembling more layers in a CNN network while improving the network’s learning. Since the ResNet-50 architecture forms a vector of 2048 features in the Fully Connected layer, and the proposed method attempts to classify three classes of Leukemia, it is necessary to modify the MLP classifier layer. It has been proposed two different configurations: One going from 2048 to 1024-512-3 (Medium) and the other from 2048 to 3 (Linear). The objective behind the different classifiers configurations is based on the assumption that adding more hidden layers is needed to approximate the feature function of each class, leading to a classification improvement.

To find the best classifier for this problem, eight models were trained based on ResNet-50, changing the activation function, the number of hidden layers and neurons in the MLP classifier, and the input images, Segmented and NoSegmented, as is shown in Table 1:

### 3.5. Visual Explainability

A crucial component of our proposed method is the integration of a visual explainability stage, which aims to provide insights into the network’s learning process and ensure that the regions of interest (ROIs) are accurately identified during Deep Learning classification. This step enhances the method’s overall effectiveness and enables clinicians to interpret the results generated by the network. Since, in the field of Deep Learning interpretability, there is currently no consensus on the best metrics-based approach for activation map generation, and it is known that each method could highlight different regions. Therefore, we perform a comprehensive analysis of four gradient-based methods, namely GRAD-CAM [34], Grad CAM++ [35], HiRes-CAM [36], and XGrad-CAM [37]. By examining these methods’ outputs, we ensure a robust evaluation of the network’s attention and activation patterns. This approach enables us to better understand the network’s decision-making process and further strengthens the interpretability of our proposed method.

### 3.6. Clustering Space Analysis

**Figure 3:**
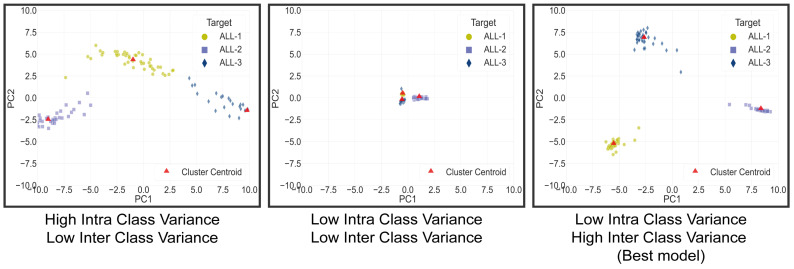
Model clustering space comparison.Where the best model is the one that enhances inter-class distance and reduces intra-class separability.

We introduced a clustering space analysis to visualize class predictions in the proposed method to enhance reliability and robustness. By obtaining the logits of each sample in the test set and their corresponding true targets, a 3-dimensional map was generated where the coordinates represented class predictions (L1, L2, and L3). Principal Component Analysis (PCA) was applied to reduce dimensionality and visualize clusters. This visualization technique allowed us to observe how the network grouped classes in the logits space, aiming to maximize inter-class variance and minimize intra-class variance. The analysis included calculating the Euclidean distance between cluster centroids to measure inter-class variance and using the standard deviation of “PC1” and “PC2” within each cluster to quantify intra-class variance. Recognizing the significance of inter-cluster distance in class prediction, we introduced the Dist/SD Ratio, a weighted ratio of 3-1 Distance/SD intra-class. We think that models amplifying this ratio may exhibit superior robustness when clustering-classifying new data, reflecting better class separability and tighter intra-class clustering for enhanced generalization performance, as is shown in Figure 3.

## 4. Results and Discussion

### 4.1. Segmentation Results

The handcrafted segmentation method was implemented using a PC, with an Intel Core i7-4510U processor, 8 GB RAM, the operating system Windows 64-bit, using Python version 3.9.7 and the libraries Scikit-image[38] and OpenCV [39]. The deep-learning segmentation was made in a Google Colab environment, using a Tesla T4 GPU, Pytorch v1.12.1 [40], Scikit-learn [41] and Monai [42]. A 10-fold cross-validation was used to assess the predictive performance of the proposed model. The dataset was randomly shuffled and divided into 10 equal parts or folds. During each iteration, nine of these folds were used for training the model, while the remaining fold was reserved for testing. This process was repeated 10 times, with each fold as the test set once. The model’s performance was evaluated on diverse data by rotating the test set across different folds improving reliability, [43,44]. Furthermore, we applied data augmentation techniques on the fly [45], such as VerticalFlip, HorizontalFlip, RandomRotate90, Transpose with a probability of *p* = 0.5 and RandomGamma, CLAHE, GaussNoise with *p* = 0.2 and a Resize (256,256) with *p* = 1. General hyperparameters were: Adam optimizer, unified focal loss, and ReduceLROnPlateau.Specific hyperparameters, for each UNet such as lambda, delta, gamma, learning rate, dropout probability, and weight decay, can be seen in Table A2.

**Figure 4:**
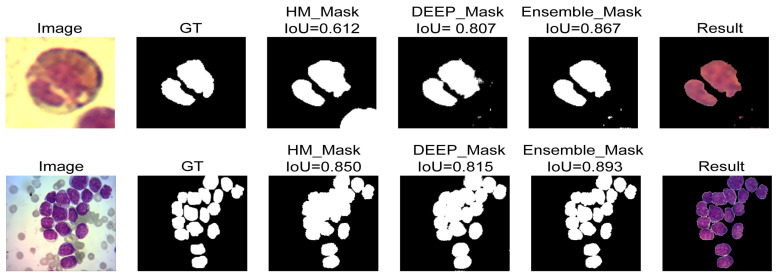
Comparison between HM, DEEP, and Ensemble results.

The proposed WBC nuclei segmentation method was evaluated in Leukemia Dataset, CellaVision, JTSC, SMC_IDB, Raabin_WBC, and ALL_IDB2 datasets. Figure 4 compares the three proposed methods on two images, one of JTSC and one from the Leukemia dataset. It can be seen that the combination of both methods, Handcraft and Deep, results in improved segmentation, even with the color differences or cell numbers in the images.

**Figure 5:**
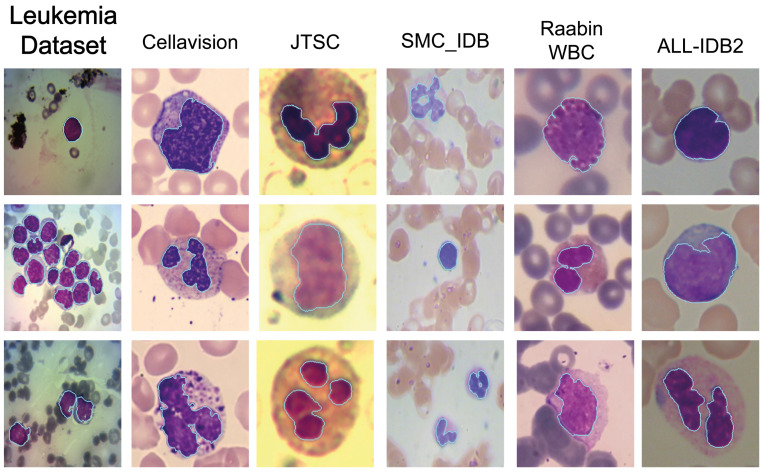
Qualitative results of the ensemble segmentation method on the six datasets. Cyan color borders the segmented nuclei.

Further perceptual results of the Ensemble method are shown in Figure 5, where a cyan border surrounds the segmented WBC nuclei. From the figure, one can perceive the overall accuracy of the segmentation method, despite the differences in saturation, color, transparency of the cells, etc.

Meanwhile, the quantitative results were obtained by comparing segmented images against their GT. Seven different quality metrics were used to assess the performance of the proposed methods. In Table 2, it can be seen that the proposed method obtains competitive results for all the databases and all the proposed quality metrics. These high-performance results confirm the robustness of the proposed segmentation system, where this system appears to demonstrate minimal variability in the output results despite changes in the input.

Moreover to general results, the proposed system is explicitly compared using each of the databases and against recent state-of-the-art methods. The results derived from these comparisons can be seen in Table 3 and Table 4.

**Figure 6:**
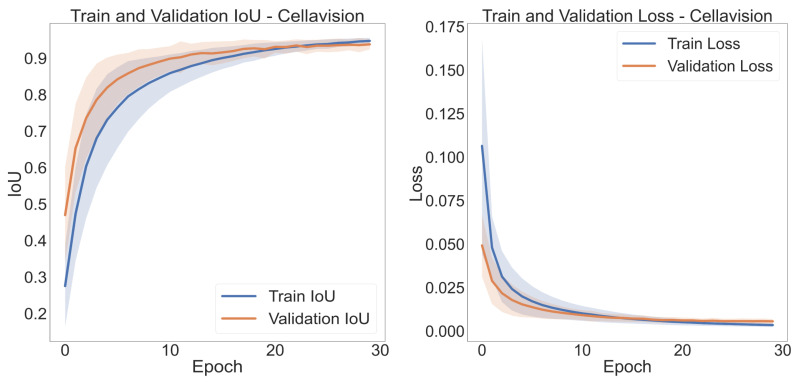
Training and Validation Intersection Over Union and Loss curves for the 10 folds.

**Figure A1:**
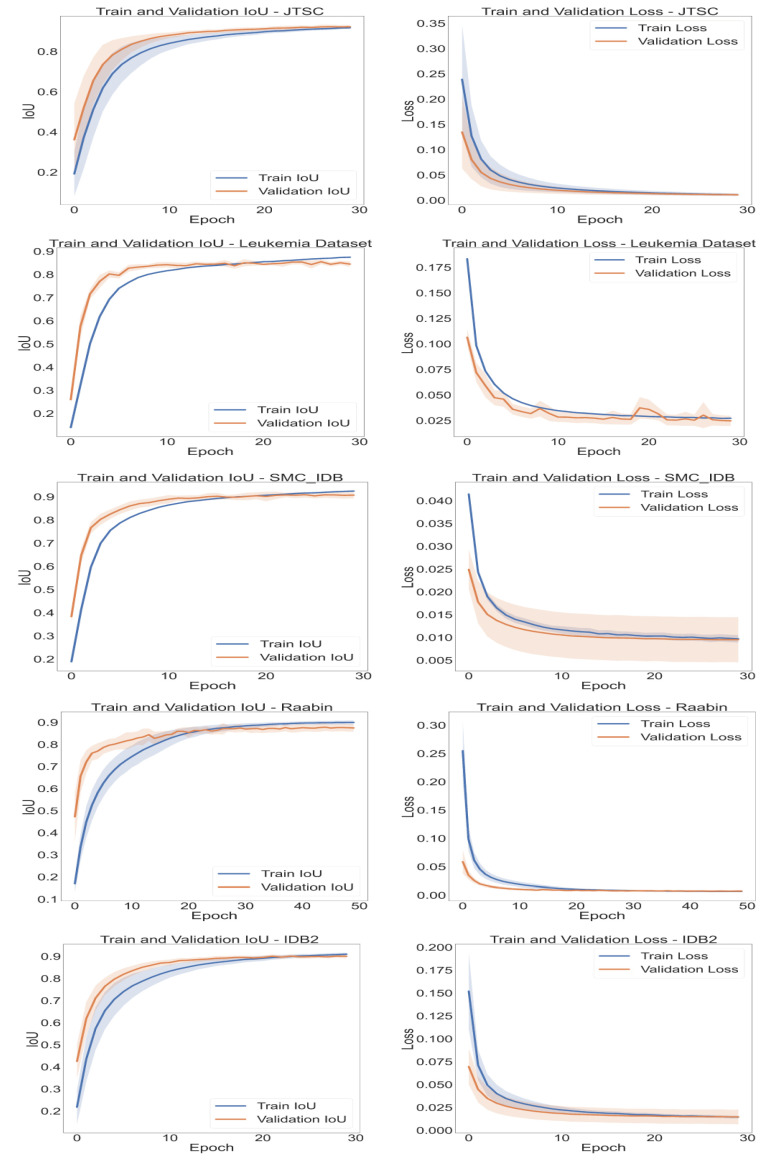
Intersection Over Union and Loss curves over 30 epochs and for each of the 10 folds.

The training and validation plots for each fold were obtained for evaluating the adequate training of each U-Net model, as shown in Figure 6. From these graphs, it is possible to observe the correct network learning for Cellavision and the other databases. The rest of the curves can be found in Figure A1.

### 4.2. Leukemia Classification

For the classification stage of the method, previously segmented images from the Leukemia Dataset were used for the **Segmented** Models and Original Images for the **Non-Segmented** Models. Both datasets were divided into a 90% Train-Validation split and a 10% Test split. A stratified K-fold with 10 folds was then applied to the Train-Validation Split. Each model was trained for 30 epochs during each K-fold. For each training set in the K-fold, ‘on the fly’ data augmentation operations were applied, including Vertical Flip, Horizontal Flip, RandomRotate90, Random Gamma, CLAHE, Transpose, and Gaussian Noise, each with a probability of *p* = 0.5. Finally, all the images were transformed with Resize (232), CenterCrop (224), and Normalize (mean = (0.485, 0.456, 0.406), std = (0.229, 0.224, 0.225)). The hyperparameters of the ResNet-50 models included a batch size of 8, a learning rate of 1 $\times \;10^{-5}$, an Adam optimizer with a weight decay of 1 $\times \;10^{-4}$, cross-entropy loss, and the ReduceLROnPlateau learning rate scheduler.

**Figure A2:**
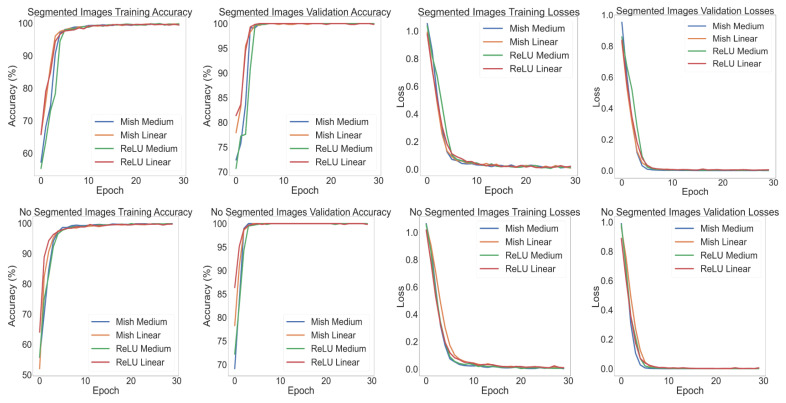
Average accuracy and loss curves for the eight proposed models with 10 K-fold.

The top four results from the ten K-fold validations across the eight models are presented in Table 5, while the corresponding training and validation plots can be found in Figure A2. These results provide evidence for the accuracy of the proposed classifier.

Comparing our method with six classifiers used by Ochoa-Montiel et al. for the Leukemia Dataset reveals that deep-learning-based methods, such as LeNet, AlexNet, and our proposed method, yield superior results in contrast to handcrafted methods such as MLP and Random Forest (see Table 6). Our study presents methods that are competitive within this landscape. However, as outlined in the Related Work (Section 1) and Methods (Section 3) sections, we go a step further by extending our analysis beyond conventional metrics. We incorporate Explainable AI (XAI) and clustering space analysis to affirm the robustness and reliability of our model [5,7].

### 4.3. Clustering Space Analysis Results

In this phase, 10% of the hold-out datasets were used to test the robustness of the model. Transformations commonly found in real environments [46], such as MotionBlur (blur_limit = 5), MultiplicativeNoise, GaussNoise (var_limit = 10, mean = 0), were applied to each image, in addition to the transformations mentioned in Section 4.2. Table 7 demonstrates that the two most robust models, yielding the best metrics, are those trained on segmented images, specifically with the Mish and ReLU activations, respectively. In contrast, the models most sensitive to daily noise are those trained on raw, non-segmented images.

**Figure 7:**
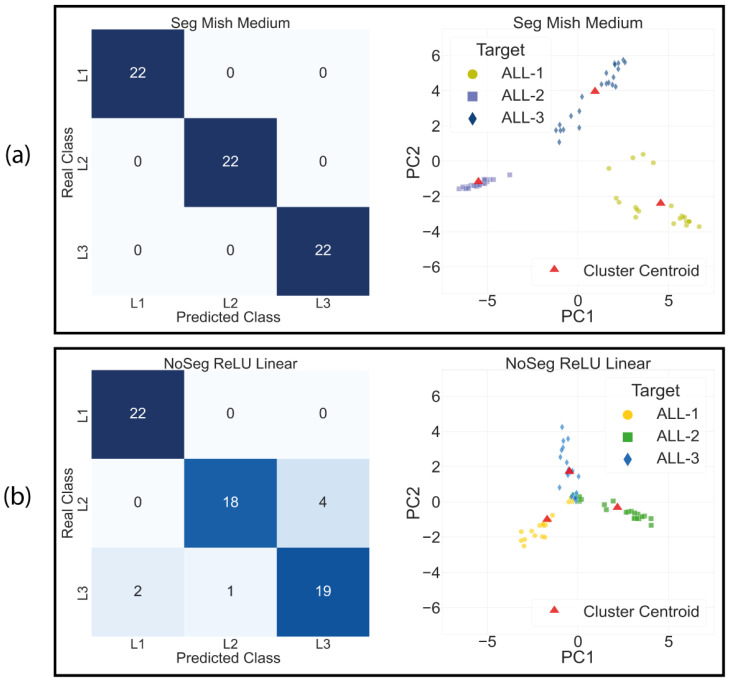
Clustering comparison between the best model Segmented Mish Medium in (**a**) and the worst model in No Segmented ReLU Linear (**b**).

On the other hand, the results of the clustering analysis, shown in Table 8, indicate that the two best models, those that improve inter-class separability and decrease intra-class separability, are the segmented models with Mish and ReLU activations. In contrast, the models with the poorest clustering results are the unsegmented ones. The visual results from the aforementioned tables are presented in Figure 7. Here, the ’Segmented Mish Medium’ model, shown in Figure 7a, performs the best in clustering and achieves higher separability, suggesting that it is learning features that better differentiate the classes. Conversely, the ’NoSegmented’ model, shown in Figure 7b, has lower intra-class separability, making classification more difficult. This leads to the classification results that can be appreciated at their respective confusion matrix.

### 4.4. Class Activation Maps

**Figure 8:**
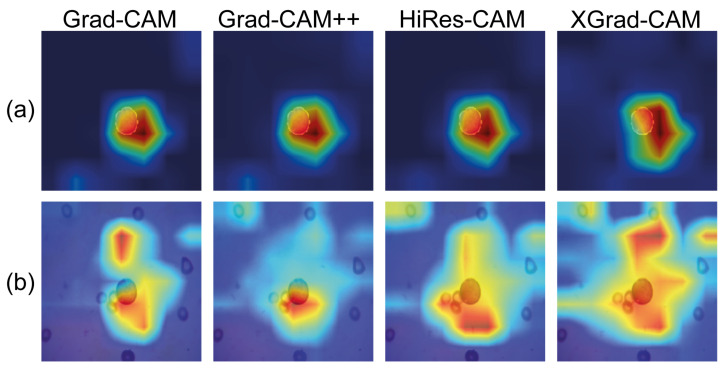
Comparison of Gradient Class Activation Maps between Segmented Mish Medium in (**a**) and NoSegmented ReLU Linear in (**b**). Red highlighted areas indicate more attention, while deep blue areas mean null attention.

The class activation maps for the ’Segmented Mish Medium’ and ’NoSegmented ReLU Linear’ models are shown in Figure 8. From these, it is apparent that applying segmentation to the WBC images, as shown in Figure 8a, allows the network to focus precisely where the WBC kernels are located. Conversely, in Figure 8b, the network is easily distracted due to the shared similarities between the WBC and blood cell characteristics.

**Figure 9:**
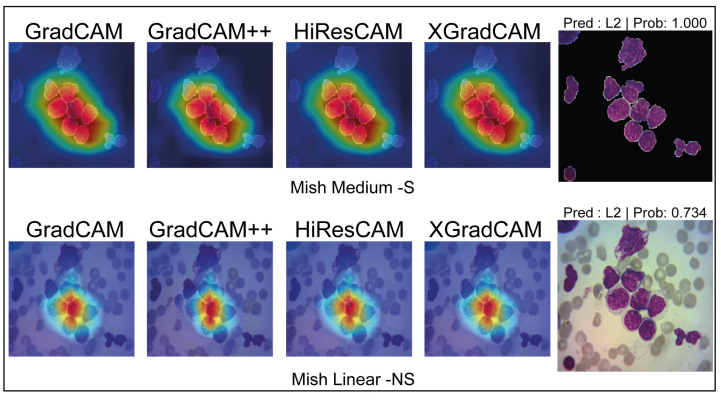
Comparison of Gradient Class Activation Maps between Segmented Model Mish Medium and NoSegmented Mish Linear. Red highlighted areas indicate more attention, while deep blue areas mean null attention.

By employing various activation maps, we can discern the semantic connections inferred by the network for classification. This is illustrated in Figure 9, where the network makes two distinct semantic associations from the same images in the Test Dataset, both leading to correct classifications. The segmented image model accurately classifies L3 with a high confidence level of 0.999, attributable to the model’s focus on the WBC. On the other hand, the ‘NoSegmented’ model also correctly classifies L3 but with a reduced confidence level of 0.734, indicating that the model may be making associations atypical to L3. For additional results, see Table A3.

**Figure 10:**
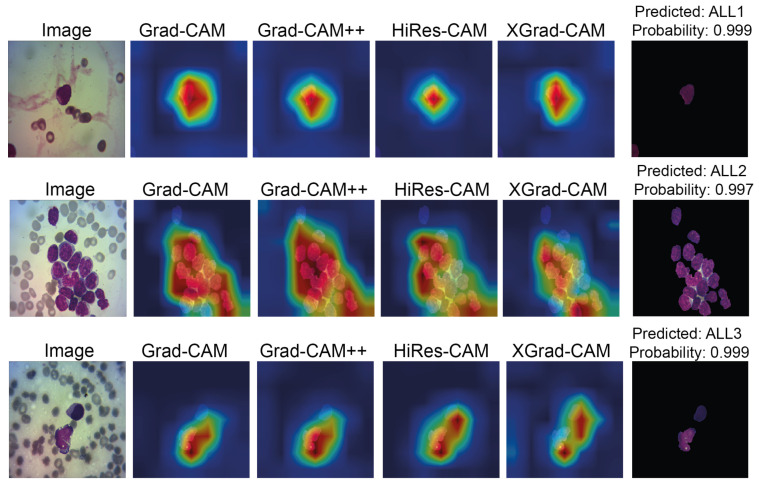
Visual explainability and classification results. The input image is segmented to enhance ResNet-50 attention; then, the image is classified with high accuracy. Red highlighted areas indicate more attention, while deep blue areas mean null attention.

Finally, based on the previous results, the ‘Segmented Mish Medium’ model emerged as the best overall for classification, as it improves both classification performance and explainability. Summary results from our proposed method can be found in Figure 10.

### 4.5. Discussion

Our experimental results underscore the advantages of integrating a highly accurate handcrafted segmentation algorithm with deep-learning-based segmentation. This combination has proven to significantly enhance the classification process. Employing a pre-segmentation approach as a hard attention mechanism prior to the classification of a Leukemia Dataset not only improves the quantitative outcomes but also enhances the model’s explainability. Furthermore, segmented models have demonstrated the capability to direct greater attention to the Region of Interest (ROI) for white blood cells (WBCs). The fusion of these methodologies significantly boosts model interpretability and reliability through the attention mechanism and visual explanation. It also paves the way for analyzing the logit space generated by the models through cluster space analysis. This could provide measures of class separability and indirectly assess the model’s ability to extract high-quality deep features that enhance classification.

This integrated segmentation approach could help to improve the segmentation and differentiation of cytoplasm in various cells and could be a valuable preprocessing step for classifying other malignancies.

Despite the promising results produced by our methodology, there is room for further enhancement. The need for labeled images, while necessary, is time-consuming and prone to errors. Future research could address these challenges by exploring unsupervised or deep reinforcement learning. Additionally, the incorporation of modern diagnostic techniques, such as Flow cytometric immunophenotyping, into morphology-based studies could lead to a more comprehensive and robust diagnostic tool by combining genetic and morphological characteristics.

## 5. Conclusions

In this research, we developed a novel Computer-Aided Diagnosis (CAD) system for Acute Lymphoblastic Leukemia (ALL) classification. This innovative system utilizes an ensemble of state-of-the-art white blood cell segmentation techniques, functioning as a hard attention mechanism, and has achieved a remarkable Intersection over Union (IoU) of 0.91 across six databases. Our ResNet-50 model, equipped with the hard attention mechanism provided by the white blood cell segmentation, demonstrated enhanced performance. Furthermore, we ensured greater transparency by incorporating visual Grad CAM interpretation and clustering analysis. The developed CAD system represents a significant step forward in improving the accuracy of ALL diagnoses, potentially leading to better patient outcomes.

In terms of future work, we plan to expand our model to classify various types of white blood cells and synergize image and genetic data to create a more powerful ensemble classifier.

## Figures and Tables

**Table 1:** Summary of the different modifications in the developed models.

Input Image	
**S**egmented	Train the model with the previously Segmented Images
**N**o**S**egmented (Ablation)	Train the model with the original images (No Segmented Images) (Traditional manner)
**MLP Classifier**	
Linear	Modify the MLP classifier from 2048 to 3 neurons
Medium	Modify the MLP classifier from 2048 to 1024-512-3 neurons
**Activation Function**	
Mish	Change all the activation functions of the model to Mish, including MLP classifier
ReLU	Change all the activation functions of the model to ReLU, including MLP classifier

**Table 2:** Results of the proposed Ensemble method for the WBC datasets.

Dataset	Acc (%)	Pre (%)	Rec (%)	Spec (%)	DSC (%)	IoU
Leukemia Dataset	98.50	88.32	95.03	98.59	91.16	0.840
CellaVision	99.32	97.08	97.88	99.57	97.40	0.951
JTSC	99.03	96.38	96.09	99.50	96.10	0.926
SMC_IDB	99.62	95.57	96.30	99.81	95.78	0.920
Raabin_WBC	98.99	97.38	94.71	99.65	94.83	0.923
ALL_IDB2	98.51	93.45	97.14	98.60	95.14	0.910
**AVERAGE**	**99.00**	**94.77**	**96.19**	**99.28**	**95.69**	**0.917**

**Table 3:** Leukemia Dataset WBC nuclei segmentation results. The best results are in bold, and the second best is underlined.

Leukemia Dataset						
**Method**	**Acc (%)**	**Pre (%)**	**Rec (%)**	**Spec (%)**	**DSC (%)**	**IoU**
Proposed HM	97.96	82.43	**97.63**	97.70	89.01	0.806
Proposed DEEP	98.30	85.82	95.81	98.30	90.02	0.823
**Proposed Ensemble**	**98.50**	**88.32**	95.03	**98.59**	**91.16**	**0.840**

**Table 4:** WBC nuclei segmentation comparison. The best results are in bold, and the second best is underlined.

Cellavision							
**Method**	**Acc (%)**	**Pre (%)**	**Rec (%)**	**Spec (%)**	**DSC (%)**	**IoU**	**# Test Images**
Vogado et al. [10]	98.77	97.88	**99.75**	89.39	93.22	0.873	100
Makem & Tiedeu [11]	99.37	97.37	96.97	-	97.06	0.945	100
CPNNHSV [18]	99.2	94.86	97.31	99.41	96.31	0.929	100
Makem et al. [14]	**99.43**	97.31	97.60	**99.61**	97.35	0.950	100
LDS-NET [17]	-	98.48	95.91	-	97.18	0.945	20
LDAS-NET [17]	-	**99.09**	97.11	-	**98.09**	**0.963**	20
**Proposed Ensemble**	99.32	97.08	97.88	99.57	97.40	0.951	100
**JTSC**							
**Method**	**Acc (%)**	**Pre (%)**	**Rec (%)**	**Spec (%)**	**DSC (%)**	**IoU**	**# Test Images**
Vogado et al. [10]	97.13	93.55	**98.99**	83.18	87.68	0.781	300
Makem & Tiedeu [11]	97.29	91.01	93.12	-	90.79	0.843	300
VarRGB [18]	98.38	91.10	96.29	98.68	93.88	0.885	300
Makem et al. [14]	97.79	93.64	97.60	98.43	93.17	0.884	300
Mayala & Haugsøen [15]	-	94.89	95.30	99.31	94.81	0.903	300
LDS-NET [17]	-	**98.85**	92.39	-	95.56	0.917	60
LDAS-NET [17]	-	94.42	98.36	-	**96.35**	**0.931**	60
**Proposed Ensemble**	**99.03**	96.38	96.09	**99.50**	96.10	0.926	300
**SMC_IDB (BloodSeg)**							
**Method**	**Acc (%)**	**Pre (%)**	**Rec (%)**	**Spec (%)**	**DSC (%)**	**IoU**	**# Test Images**
Vogado et al. [10]	99.15	80.51	94.51	99.30	86.46	0.761	367
Makem & Tiedeu. [11]	99.63	92.99	**97.06**	-	94.75	0.902	367
Makem et al. [14]	97.67	91.27	96.93	97.82	93.48	0.883	367
**Proposed Ensemble**	**99.62**	**95.57**	96.30	**99.81**	**95.78**	**0.920**	367
**Raabin**							
**Method**	**Acc (%)**	**Pre (%)**	**Rec (%)**	**Spec (%)**	**DSC (%)**	**IoU**	**# Test Images**
U-Net ++ [19]	-	95.98	**98.73**	-	**97.19**	**0.945**	250
Attention U-Net [20]	-	94.78	98.50	-	96.33	0.929	250
Mask R-CNN [21]	-	8.59	96.80	-	91.98	0.852	250
Mousavi et al. [12].	-	93.62	98.27	-	95.42	0.912	250
Tavakoli et al. [13].	-	**99.72**	95.26	-	96.75	0.936	250
**Proposed Ensemble**	**98.99**	97.38	94.71	**99.65**	94.83	0.923	1145
**ALL_IDB2**							
**Method**	**Acc (%)**	**Pre (%)**	**Rec (%)**	**Spec (%)**	**DSC (%)**	**IoU**	**# Test Images**
Vogado et al. [10]	**98.59**	91.24	**98.09**	98.62	94.17	0.890	300
CPNNHSV.[18]	98.32	91.59	96.11	**98.66**	93.42	0.877	300
**Proposed Ensemble**	98.51	**93.45**	97.14	98.60	**95.14**	**0.910**	300

**Table 5:** Best results for the train-validation 10 K-fold.

Model	Acc (%)
Segmented Mish Medium	99.99
NoSegmented ReLU Medium	99.97
Segmented Mish Linear	99.97
No Segmented ReLU Linear	99.97

**Table 6:** Performance Comparison for ALL Classification.The best results are in bold, and the second best is underlined.

Method	Validation Accuracy (%)
Random_Forest Set_Full. [16]	97.08
LeNet. [16]	98.36
AlexNet. [16]	99.98
**Proposed Method**	**99.99**

**Table 7:** Best and worst models in Test Dataset. The best results are in bold, and the second best is underlined.

Test Dataset				
**Architecture**	**Acc (%)**	**Pre (%)**	**Rec (%)**	**F1 (%)**
**Segmented Mish Medium**	**100**	**100**	**100**	**100**
Segmented ReLU Medium	98.50	98.60	98.50	98.50
No Segmented ReLU Linear	89.40	89.70	89.40	89.40
No Segmented Mish Medium	80.30	87.60	80.30	80.30

**Table 8:** Prediction Cluster Analysis on the test dataset. The best separability results are bold, and the second best is underlined.

	Dist L1–L2	Dist L2–L3	Dist L1–L3	Dist Total	SD Cluster L1	SD Cluster L2	SD Cluster L3	SD Total	Ratio Dist/SD
**Segmented Mish Medium**	**10.12**	**8.22**	**7.31**	**25.65**	2.05	**0.68**	2.06	2.99	**25.78**
Segmented ReLU Medium	6.60	5.17	3.65	15.41	1.09	1.43	0.66	1.92	24.12
NoSegmented ReLU Linear	3.94	3.34	2.99	10.27	1.32	1.60	1.33	2.46	12.52
NoSegmented Mish Linear	3.15	2.08	1.88	7.11	**0.94**	1.49	**0.50**	**1.83**	11.63

## Data Availability

The code presented in this study shall be made available upon reasonable request to the corresponding author for academic purposes.

## Appendix A. Segmentation and Classification Parameter’s and Extra Results

### Appendix A.1. WBC Nuclei Segmentation

The parameters for each technique that makes up the handcraft segmentation method are presented in Table A1.

**Table A1:** Handcrafted segmentation parameters.

Technique	Parameter
Bilateral Filter (Kernel size)	9
Bilateral Filter (radial, spatial sigma)	50
Closing Kernel	3
Area Filter	150
Dilation	2
Dilation (Raabin-Basophil)	4

### Appendix A.2. WBC Deep Learning Segmentation

The hyperparameters for each employed U-Net that conformed the Deep Learning segmentation phase are listed in Table A2.

**Table A2:** U-Net Train Parameters for each dataset.

U-Net Train Parameters						
**Dataset**	**Lambda**	**Delta**	**Gamma**	**Learning Rate**	**Dropout**	**Weight Decay**
Cellavision	0.4	0.7	1.0	1 × 10^−4^	0.1	1 × 10^−3^
JTSC	0.5	0.8	1.0	1 × 10^−4^	0.05	1 × 10^−4^
SMC_IDB	0.6	0.7	1.0	1 × 10^−4^	0.05	1 × 10^−3^
Raabin_WBC	0.4	0.7	1.0	1 × 10^−4^	0.5	1 × 10^−2^
ALL-IDB2	0.4	0.7	1.0	1 × 10^−4^	0.1	1 × 10^−4^
Dataset Leukemia	0.4	0.4	1.0	1 × 10^−4^	0.05	1 × 10^−5^

Learning curves for the deep-learning segmentation procedure with a 10 K-Fold. Left column shows the IoU and at the right column the loss curve.

### Appendix A.3. Deep Learning Classification

Deep-learning classification curves for the eight proposed models are presented in Figure A2. On the left column, Train and Validation Accuracy over epochs, and on the right column, Train and Validation Loss.

Table A3 shows the nine results of “accurate” classification from ALL with four models. Although all the images were correctly classified, the classification certainty differs between them.

**Table A3:** Leukemia class prediction probabilities of nine correct diagnoses. Most reliable results are in bold and second best results are underlined.

Class Prediction Probability					
	**Image**	**Mish Linear-S**	**Mish Linear-NS**	**Mish Medium-S**	**Mish Medium-NS**
ALL-1	1_1_7	0.998	0.426	**1.000**	0.945
	1_3_132	0.992	0.430	**0.999**	0.993
	1_3_158	0.998	0.464	**1.000**	0.993
ALL-2	2_1_33	0.998	0.750	**1.000**	0.991
	2_2_127	0.999	0.734	**1.000**	0.990
	2_3_202	0.999	0.778	**1.000**	0.970
ALL-3	3_1_3	0.973	0.570	**0.997**	0.846
	3_1_32	0.993	0.439	**0.999**	0.806
	3_2_11	0.998	0.492	**1.000**	0.838
**Average**		0.994	0.565	**0.999**	0.930
